# Source connectivity patterns in the default mode network differ between elderly golf-novices and non-golfers

**DOI:** 10.1038/s41598-023-31893-1

**Published:** 2023-04-17

**Authors:** J. K. Gowik, C. Goelz, S. Vieluf, F. van den Bongard, C. Reinsberger

**Affiliations:** Department of Exercise and Health, Institute of Sports Medicine, Paderborn University, Warburger Straße 100, 33098 Paderborn, Germany

**Keywords:** Cognitive ageing, Network models, Electroencephalography - EEG

## Abstract

Learning to play golf has high demands on attention and therefore may counteract age-related changes of functional brain networks. This cross-sectional study compared source connectivity in the Default Mode Network (DMN) between elderly golf novices and non-golfers. Four-minute resting-state electroencephalography (128 channels) from 22 elderly people (mean age 67 ± 4.3 years, 55% females) were recorded after completing a 22-week golf learning program or after having continued with normal life. Source connectivity was assessed after co-registration of EEG data with native MRI within pre-defined portions of the DMN in the beta band (14–25 Hz). Non-golfers had significantly higher source connectivity values in the anterior DMN compared to non-golfers. Exploratory correlation analyses did not indicate an association to cognitive performance in either group. Inverse correlations between a marker of external attention with source connectivity of the anterior DMN may suggest a trend in the golf group only, but have to be replicated in future studies. Clinical relevance of these findings remains to be elucidated, but the observed difference in the anterior DMN may provide a starting point to further investigate if and how learning golf may have an impact on physiological age-related cognitive changes.

## Introduction

Aging is accompanied by structural and functional changes of the brain. Besides grey and white matter atrophy^1–3^, pathological mechanisms in the context of Alzheimer’s disease (AD) such as cerebral hypometabolism^4,5^ as well as amyloid-ß and tau deposits^6^ have the potential to impair functional network organization^7,8^.

Pathological mechanisms related to AD were also found in elderly people experiencing subjective memory complaints (SMC)^9,10^. It was further associated with an increased risk for AD compared to elderly people without SMC^11^. The etiology of SMC, however, is heterogeneous, and can also be caused by other factors not related to AD, such as psychiatric or chronic diseases or psychological distress^12^. Nevertheless, SMC due to preclinical AD were shown to be associated with impaired functional network organization^13^.

As a consequence of an alteration of functional networks, dedifferentiated activity especially of sensorimotor, visual, and dorsal attention networks were described^14^.

The Default Mode Network (DMN) as the hierarchically superior resting state network is particularly affected by functional reorganization processes in the context of aging and AD^15^. A reduced ability to deactivate the DMN during external attention demanding tasks was observed in elderly people and AD patients, which points to a stronger de-differentiated involvement of DMN hubs (grey matter regions of high significance in a network) in other sensory (sensorimotor, visual) or cognitive networks (dorsal attention, visual-spatial)^16,17^. Moreover, increases in connectivity to regions outside the DMN could be observed, which point to lower network integrity^18,19^. A reduced anterior–posterior (“core”) connectivity in the DMN has been described in elderly people^15,20^, suggesting a disconnection of the anterior and posterior portions of the DMN during aging^20^. Reduced functional connectivity was found in the posterior DMN in elderly people compared to younger adults, whereas connectivity in the anterior DMN showed no uniform pattern^15^. Interestingly, AD patients had greater declines in posterior DMN connectivity and higher functional connectivity values in the anterior DMN compared to healthy elderly people^15^.

Evidence from epidemiological studies suggests that regular physical activity has the potential to counteract age and AD-related changes by reducing amyloid burden^21^ and prevent cerebral atrophy^22–24^. Especially cardiovascular fitness and aerobic exercise interventions were investigated most frequently in this context, with positive effects on cognitive performance^25,26^ and functional integrity of the DMN^27–29^. Although it may be concluded that cardiorespiratory fitness, habitual physical activity and even acute bouts of exercise may strengthen functional connection of various brain regions and networks and at the same time may improve multiple neurocognitive subdomains, the exact underlying mechanisms still remain to be elucidated^30^.

Moreover, in addition to aerobic exercise functional networks also adapt in response to complex training, which involves higher order cognitive functions. Binder and colleagues^31^ found task-specific increased functional connectivity and efficiency in networks, which were stimulated by multi-domain cognitive training in elderly people even 1 year after the intervention. Similar practice-related structural and functional adaptations of networks were also found for musicians^32,33^, meditation practice^34^ and middle-aged golf novices^35^.

Golf is a popular leisure time activity in the elderly population with high demands on cognitive and motor functions. Learning to play golf mainly consists of performing different swings (full swing, pitch, chip, put) and golf-specific rules. In a recently published 22-week randomized trial, a significant effect of learning to play golf on attention performance was found in elderly people with SMC^36^. The findings may suggest that learning to play golf has an impact on specific cognitive domains, such as attention. The impact on functional characteristics of brain networks, such as the DMN, has not been explored and described yet. There is a need for a better understanding of functional mechanisms induced by different modes of exercise to design effective preventive interventions against cognitive decline in elderly people.

Therefore, in an explorative approach and based on cross-sectional data from the randomized trial^37^, this study aimed to compare functional connectivity of the DMN and the association to cognitive performance in elderly people with subjective memory complaints (SMC) and a history of 22-week golf training to a control group without golf-experience that continued with daily life. Such knowledge may contribute to understand the relationship between complex training, which is not cardiorespiratory in nature, induced functional connectivity changes of brain networks and associated cognitive effects, but this study should be seen as hypothesis generating. Thus the exploration of functional characteristics of the DMN was approached with two different methods:

First, by comparing DMN source connectivity between both groups. Considering that aging and AD affects subsystems of the DMN differently and that we were interested in domain-specific functional differences in elderly golf-novices, we divided the DMN in our analysis into anterior, posterior and “core” portions.

Second, there is evidence from simultaneous resting-state EEG and functional magnetic resonance imaging (fMRI) studies indicating a negative association between frontal midline theta power (oscillatory activity in the theta frequency band, 4–7 Hz) and the blood-oxygen-level-dependent (BOLD) signal of DMN regions^37,38^. It was suggested that frontal midline theta power (as a marker of external attention) may index functional antagonism to the DMN in resting-state in healthy adults^38^. The inverse relation between frontal midline theta power and the DMN may also be affected by the reorganization processes of functional networks due to age or by early preclinical neurodegenerative processes of AD, but this has not been described specifically yet. The investigation of a potentially reverse relationship between frontal theta as a neurophysiological marker of external attention and DMN source connectivity in elderly people with SMC may provide the basis for further studies investigating mechanisms of age-related dedifferentiation of brain networks (including the DMN).

## Results

In this study, we analyzed cross-sectional data from 22 participants, of which 12 were golf novices, who completed a golf training 3 times per week over 22-weeks, and 10 non-golfers, which served as a control group. All participants reported subjective memory complaints, but had no diagnosed mental or neurological disease (e.g. depression, AD, Parkinson’s Disease). The Alzheimer’s Disease Assessment Scale Cognitive Subscale (ADAS-Cog^39^, revealed normal age-appropriate scores for the majority of the participants (n = 23, scored between 1 and 9 points). Four participants scored between 10 and 13 points, which possibly indicates mild cognitive impairment^40,41^. Two of them were in the golf-novices group, and two were in the non-golfer group. In general, both groups were comparable regarding age, gender, education, and ADAS-Cog score, and daily activity habits indicated by the Physical Activity Scale for Elderly (PASE) (p > 0.05). Of note, there was a tendency towards better performance on the 6-Minute-Walk Test (6MWT) in the control group which was not significant (p = 0.1). Baseline characteristics are presented in Table 1.

**Table 1:** Baseline characteristics of the participants, presented as means with standard deviation, except for gender and handedness.

	Golf-Novices	Non-Golfer	p-value
n	12	10	
Gender			
Male	6	4	
Female	6	6	
Age (years)	67 (4.79)	68 (3.77)	0.842
Education (years)	15.17 (4.86)	12.40 (3.86)	0.165
ADAS-Cog (score)	6.75 (2.26)	6.60 (3.27)	0.789
6MWT (m)	625.94 (168.99)	699.60 (131.14)	0.102
PASE (score)	158.79 (49.96)	143.65 (47.95)	0.364
BMI	26.70 (3.76)	25.27 (2.62)	0.291
handedness (n)	Left-handed: 1	Left-handed: 0	

Group differences were tested with the Mann–Whitney U test.

*ADAS-Cog* Alzheimer’s disease Assessment Scale-Cognitive Subscale, *BMI* Body Mass Index, *PASE* Physical Activity Scale for Elderly, *6MWT* 6-min-walk test, *BMI* body-mass-index.

Four-minute high-density resting-state EEG recordings were collected from each participant and frontal midline theta power was extracted via independent component analysis. The data was further co-registered with the individual (native) MRI of each participant, and DMN activity was assessed by phase locking value (PLV)-analysis of source signals^42^. Source connectivity of DMN hubs was calculated in the beta frequency band (14–25 Hz) based on Kabbara et al.^42^. Mean connectivity values of the whole DMN and DMN portions (anterior DMN, posterior DMN and core DMN) were used for correlation analyses with frontal midline theta power as well as for the group comparisons. Bonferroni correction was applied for all analyses.

### Group differences in the DMN and for cognitive performance

Significant group differences were found for anterior DMN mean source connectivity, which was higher in the control group (golf 0.41 ± 0.07 vs. control 0.52 ± 0.06, p = 0.028). Similarly, mean strength of anterior DMN was significantly higher in the control group (golf 2.49 ± 0.41 vs. control 3.17 ± 0.39, p = 0.028). No significant group differences were found for other connectivity and graph measures of the DMN as well as for cognitive functions.

An overview of the results is presented in Table 2.

**Table 2:** Group differences were examined with the Mann–Whitney-U Test at a significance level of p < 0.05.

	Golf-novices (n = 12)	Non-golfer (n = 10)	p-value
Corsi forward (sequ)	7.91 (2.43)	7.60 (2.37)	0.815
Corsi backward (sequ)	6.83 (2.17)	7.50 (3.06)	0.443
INHIB reaction time (s)	0.37 (0.05)	0.34 (0.05)	0.248
INHIB correct responses (%)	94.58 (3.47)	91.90 (7.12)	0.425
TMT A (s)	26.58 (5.47)	27.50 (9.82)	0.817
TMT B (s)	48.15 (13.07)	52.72 (30.05)	0.843
Beta DMN PLV	0.27 (0.03)	0.29 (0.03)	0.107
Beta DMN strength	3.79 (0.41)	4.04 (0.39)	0.123
Beta aDMN PLV	0.41 (0.07)	0.52 (0.06)	0.028*
Beta aDMN strength	2.49 (0.41)	3.17 (0.39)	0.028*
Beta pDMN PLV	0.43 (0.07)	0.47 (0.07)	0.123
Beta pDMN strength	1.28 (0.21)	1.33 (0.31)	0.821
Beta core DMN PLV	0.32 (0.13)	0.41 (0.12)	0.107
Beta core DMN strength	0.82 (0.28)	0.83 (0.28)	0.628

Bonferroni-corrected p-values are reported. Variables are presented as means with standard deviation.

*INHIB* response inhibition, *TMT* trail making test, *DMN* default mode network, *PLV* phase locking value, *aDMN* anterior default mode network, *pDMN* posterior default mode network, *core DMN* caudal anterior cingulate cortex and posterior cingulate cortex.

Significant values are given in bold.

Exploratory correlation analyses between DMN source connectivity and cognitive performance showed no significant associations in either group after Bonferroni correction.

### Frontal midline theta power and the DMN

**Figure 1:**
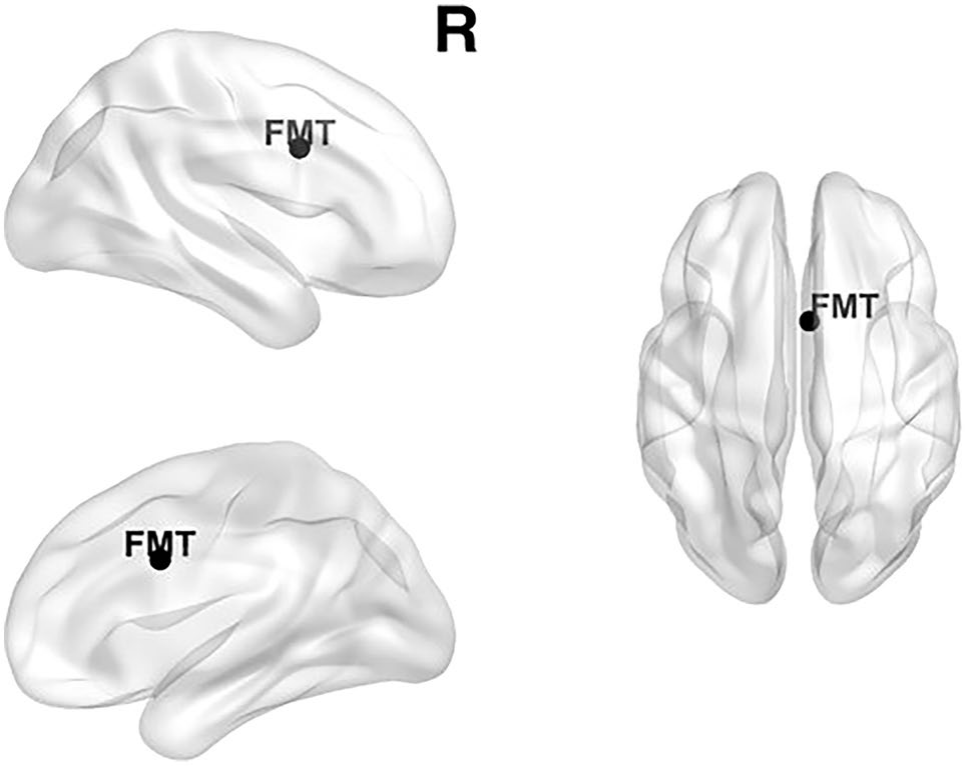
Localization of frontal midline theta (FMT, 4–7 Hz) based on the 22 individual components. MNI coordinates: X = 4.2 Y = 7.9 Z = 30.8. The brain networks were visualized with the BrainNet Viewer^70^.

The dipole of the average independent theta components (n = 22) was localized in Brodmann area 24 (MNI coordinates: X = 4.2 Y = 7.9 Z = 30.8), with a goodness-of-fit of 93%. Brodmann area 24 is defined as the anterior cingulate cortex (see Fig. 1).

Frontal midline theta power was not significantly different between groups (golf: 0.04 ± 0.06 vs. control: 0.02 ± 0.02, p = 0.628).

We observed a trend towards negative correlations for anterior DMN mean connectivity and mean DMN strength with power in the golf group (r_s_ = − 0.699, p = 0.176; r_s_ = − 0.678, p = 0.240), but the values were not significant after Bonferroni-correction. No significant correlations were found between functional connectivity of the whole posterior or core DMN and frontal midline theta power in either group.

An overview of the results is presented in Table 3.

**Table 3:** Spearman correlations (r_s_) between frontal midline theta power and connectivity of the DMN (Default Mode Network) for the golf-novices (n = 12) and non-golfer group (n = 10).

Mean PLV	Golf-novices	Non-golfer
Frontal midline theta power (r_s_, p-value)		
DMN	− 0.448	0.310
	0.145	0.383
aDMN	− 0.699	− 0.182
	0.176	0.614
pDMN	− 0.182	− 0.030
	0.572	0.934
Core DMN	− 0.650	− 0.267
	0.352	0.455
Mean strength		
DMN	− 0.448	0.310
	0.145	0.383
aDMN	− 0.678	− 0.091
	0.240	0.802
pDMN	− 0.175	− 0.109
	0.587	0.763
Core DMN	− 0.490	− 0.006
	0.106	0.987

*aDMN* anterior default mode network, *pDMN* posterior default mode network, *core DMN* caudal anterior cingulate cortex and posterior cingulate cortex.

## Discussion

In this hypotheses-generating study, an EEG PLV-analysis approach on source signals with native MRI was used to explore differences of DMN source connectivity in elderly people with a history of learning golf compared to elderly non-golfers. Two different approaches were used to explore the differences: the first approach revealed a difference in mean source connectivity and strength in the anterior DMN between both groups. Non-golfers had significantly higher mean values than golf-novices. We additionally conducted an analysis of variance with 6MWT as a covariate to account for bias due to cardiovascular performance, which did not contradict our findings (p < 0.002). However, an association between cognitive performance and DMN source connectivity could not be observed in either group, which complicates a clear interpretation regarding clinical significance of the results. The high number of exploratory correlation analyses also caused that no p-value remained significant after Bonferroni correction, which may impeded the observation of such an association.

The second approach explored the relation to a parameter indexing functional antagonism to the DMN, which might be affected by age or AD-related physiological changes. Exploratory correlation analyses revealed no significant associations in either group. The findings may suggest a trend towards a negative association between anterior DMN mean source connectivity and frontal midline theta power in the golf-novices group only (p < 0.240), which should be investigated in future studies. A similar inverse association between frontal midline theta and the DMN has been described in healthy young adults in a previous study^37^. In general, the association between anterior DMN source connectivity and frontal midline theta power may have the potential to serve as a marker to investigate age-related neuronal adaptation to decreased functional specialization of brain areas.

Learning to play golf was shown to be associated with regional structural adaptations of the brain^34^. Interestingly, significant anatomical differences were only observed between golf-novices and professional golf players in the literature, but not in comparison to moderately skilled players^43^. In general, patterns of functional brain networks might differ when learning a new motor skill compared to training of rather automated movements. Learning a new motor skill, like golf, was found to be associated with higher activation in brain regions defined as the Dorsal Attention Network (DAN), a network involved in task-related attention and cognitive control^45^ that is functionally anti-correlated with the DMN^46^. This activation in the DAN decreased with automatization of movements, presumably indicating less cognitive control and attention^45^. Therefore, learning a new and complex motor skill, like golf, may induce different network changes compared to repetitive training, such as aerobic exercise. In accordance, Voss et al.^28^ found increased functional connectivity in DMN regions of elderly people engaging in a combined low intensity balance, stretching, and toning program after only 6 months of regular training, whereas elderly people in the moderate and repetitive aerobic exercise group showed increased DMN integrity after 1 year first.

Therefore, engagement in low-intensity but complex motor stimulating activities may have an influence on functional brain network organization^46,47^ beyond cardiovascular fitness and metabolic intense exercise^28^, which is usually recommended for elderly people to prevent cognitive decline. The mechanisms causing these changes might be different. Aerobic exercises, like walking, are characterized by simple, repetitive, automatic movements and were found to increase cerebral blood flow, induce several neurotrophic factors supporting synaptogenesis and angiogenesis, and result in enhanced synthesis of cerebral tissue^22,48,49^. Therefore, aerobic exercise presumably has a rather general and unspecific effect on brain network organization, which positively affects cognitive performance. Golf on the other hand might induce changes directly and more specifically depending on the training history. For instance, regular coordination exercises increased functional connectivity specifically in the visual-spatial network in elderly people^46^.

Of note, the requirements of a golf match with some of the cognitive-behavioral symptoms of early AD, including attention, visual-spatial relations and working memory functions. Adapted to the patient’s abilities, golf theoretically may serve as a potentially beneficial treatment for people at risk of AD.

However, the potential effects of learning to play golf on source connectivity patterns of the DMN remain to be established, preferably in a randomized controlled design with an appropriate sample size. Golf is one of several lifestyle-related factors influencing cognitive performance and functional network characteristics, including cardiovascular fitness^28,50^, leisure time activities like chess or playing an instrument^32^, genetic predisposition^51–53^ as well as diet^54,55^, which may have influenced our findings. The non-golfing control group did not receive additional treatment to account for the influence of other factors related to the intervention that may also had an effect on results, such as an increased social interaction. An adequate control treatment should therefore be considered in the longitudinal replication of this study.

A limitation of our study is also the small, but well-characterized sample, which prohibited the application of more powerful parametric statistical tests. Combining MRI and EEG to improve the spatial resolution of functional changes as performed in our study may further increase the sensitivity of the observed differences in the DMN despite the small sample. However, a future randomized trial may replicate findings in an adequately powered sample. The number of data samples for analysis may be few in this study, but to the best of our knowledge, there is no consensus on the optimal number yet. In addition, each epoch was carefully selected and checked for eligibility by two authors (JKG and CR), of which one is an experienced neurologist (CR). The dipole localization might be biased by its tendency to estimate the source too deep, when there is a more superficial source. However, frontal midline theta was located in or near the anterior cingulate cortex (BA 24), which is in line with previous findings^38,56,57^. Overall, the source connectivity approach reduces field spread and volume conduction problems of cortical EEG, but cannot completely diminish the effects^68^.

The findings of this study suggest different source connectivity patterns in the anterior DMN of elderly golf-novices compared to non-golfers. An association to cognitive performance could not be observed in either group and therefore, clinical relevance of these differences remains to be elucidated. We also observed a non-significant trend regarding an inverse association between frontal midline theta power and anterior DMN source connectivity in golf-novices only. The association may serve as a potential marker to investigate age-related neuronal adaption to decreased functional specialization of brain areas in future studies. Therefore, the findings of this hypotheses-generating cross-sectional study may provide a starting point to investigate potential changes in the anterior DMN after learning a new and complex motor skill, like golf, in older age. A longitudinal and preferably randomized study with a larger sample size and an adequate control treatment would provide a more detailed understanding of mechanisms induced by learning to play golf on source connectivity patterns in the anterior DMN in the elderly brain and how it may prevent cognitive decline due to AD.

## Methods

The data was collected during a 22-week randomized controlled trial from May to December 2018. Participants were recruited via local newspapers, social media advertisements, and personal contact with organizations providing leisure activities for elderly people. During the intervention, elderly people with subjective memory complaints and no prior golf-experience learned to play golf under the supervision of a professional golf trainer. The non-golfers served as a control group and continued with daily life. Both groups were asked to document daily activity habits with a questionnaire during the intervention (Physical Activity Scale for Elderly, PASE)^59^. The analyzed data set was collected immediately after the intervention. The trial was registered at the German Clinical Trials Register (DRKS00014921). The ethics committee of the “Westfälische Wilhelms-Universität Münster”, Germany, approved the study protocol according to the declaration of Helsinki. Participants were informed about the main research aims and personal data management and gave their informed consent before the start of the intervention.

### Golf training

The golf training consisted of three sessions per week, each lasting 60 min for 22 weeks. Two of the three sessions were supervised and instructed by trainers. The third session was not supervised, but participants were asked to practice the previously acquired skills independently at the driving range.

The supervised golf program included 18 practice sessions and 25 sessions at the driving range. All sessions started with a short warm-up (10 min), which consisted of coordination and stretching exercises. In the practice sessions (week 1–8), participants learned basic golf techniques, starting with putting and chipping. After 5 weeks, pitching was introduced and practiced and after 7 weeks, participants learned the full golf swing. Golf trainers gave individual feedback to improve the techniques, e.g. via videos or verbal instructions during the sessions. At week 9, participants started to practice at the driving range.

### EEG measurements

Resting-state measurements were conducted with a high-density 128-channel Electroencephalography (EEG) actiCap system from Brain Products (Brain Products GmbH, Gilching, Germany). The cap was positioned according to the international 10–10 system and impedances were constantly checked and kept below 15 kΩ. The sampling rate was set to 500 Hz. The ground electrode replaced FPz, and the reference electrode replaced FCz. Participants were measured in supine position with eyes closed in an acoustically attenuated darkened room and were instructed to relax but stay awake during the 4 min recording. Individual electrode locations were registered with the BrainVision CapTrack software (Brain Products GmbH, Gilching, Germany).

### Preprocessing and connectivity analyses of source signals

**Figure 2:**
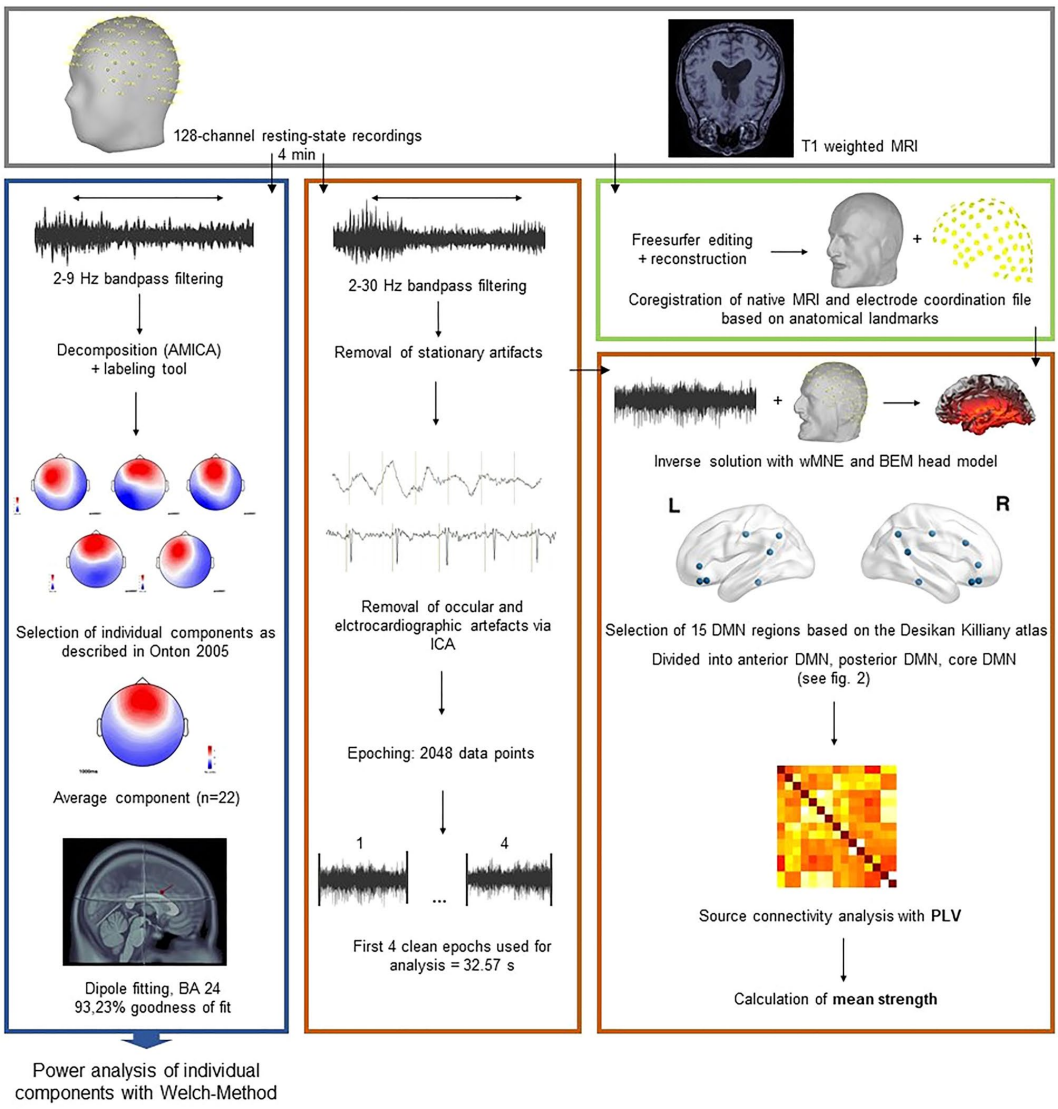
Data processing stream overview adapted from Kabbara et al.^41^. Blue box: Determination of frontal midline theta with independent component analysis. The average component was localized within Brodman Area (BA) 24. Power spectrum density (Welch method) was used to extract relative power over the whole epoch. Orange box: Preprocessing steps and selection of epochs for source connectivity and power analysis in the Default Mode Network (DMN). Green box: Preprocessing of native MRI and co-registration with the electrode coordinates. *AMICA* adaptive mixture independent component analysis, *ICA* independent component analysis, *wMNE* weighted minimum norm estimation, *BEM* boundary element model, *PLV* phase locking value.

An overview of data analysis is presented in Fig. 2.

The preprocessing was done with BrainVision Analyzer Version 2.1.2 (Gilching, Germany: Brain Products GmbH, www.brainproducts.com). The raw data was first visually screened for bad channels, and the sampling rate was downsampled from 500 Hz down to 256 Hz. Then, a Matlab-based (Matlab R2017a, Mathworks Inc., MA, USA; www.mathworks.com) screening algorithm eBridge^60^ and magnitude squared coherence values between 0.9 and 1 for visual inspection were both used to detect electrical bridges in the data. If the results from the visual inspection and the algorithm were identical, the bridged channels were interpolated with the spherical spline method as implemented in the BrainVision Analyzer software. If the identified electrode bridges of both methods were not identical, the electrodes were additionally visually inspected (difference between both channels) and interpolated, if necessary. Participants were excluded from analysis if more than 15% of all channels or the reference electrode was bridged^50^.

Then, the individual electrode coordinates were loaded into the software and the data were re-referenced to the average. Zero Phase Shift Butterworth Filters were applied, with a low cutoff at 1 Hz (time constant 0.159 s, order: 4) and a high cutoff at 30 Hz. Sinusoidal line noise was reduced by the application of a 50 Hz notch filter. Before applying independent component analysis (ICA) to the data, stationary artifacts were removed via visual inspection. Then, extended infomax ICA implemented in the BrainVision Analyzer software was used to exclude eye movements and electrocardiographic artifacts. The cleaned data was subsequently cut into epochs of 2048 data points, resulting in 8 s per epoch. For phase synchronization measures in source space, epoch lengths of at least 6 s were recommended to have stable results^61^. Each epoch was visually inspected for remaining artifacts. An experienced neurologist (CR) further screened epochs for signs of drowsiness and excluded them, if necessary. The first 4 “artefact-free” epochs of the 4 min recording were used for the PLV-analysis on source signals as recommended in^62^), resulting in an overall analysis window of 32 s per subject. The epochs were converted into ASCII files and exported to Brainstorm^63^.

Before the EEG recordings, participants were scanned with a 1.5-T MRI Scanner (Hitachi Medical Systems, Hitachi, Japan). A 3D RSSG (RF-spoiled SARGE) protocol with 170 axial slices, echo time (TE) = 2.3 ms, repetition time (TR) = 10.6, slice thickness = 1.0 mm was used. Cortical reconstruction and volumetric segmentation of the individual MRIs were performed using the automated Freesurfer stream (http://surfer.nmr.mgh.harvard.edu/). Data quality was checked for each MRI, and manual editing was applied to correct artifacts (movement artifacts and surfaces).

The PLV-analysis on source signals was conducted as described in Kabbara et al.^42^. Briefly, the MRI was co-registered with the individual electrode location file to compute a precise forward model. A cortical mesh with 642 vertices per layer (scalp, skull, brain) was computed with the Boundary Element Method (BEM) using OpenMEEG^64^. To solve the inverse problem, the weighted Minimum Norm Estimate (wMNE) approach with constrained dipole orientations and current density maps as a measure was used. 60 s of the individual resting-state recording served as a noise covariance matrix. Only the diagonal elements were saved to account for variance measured at each sensor. The reconstructed time series from 15 regions of interest (ROI), which were previously identified as DMN hubs, were selected based on the Desikan-Killiany atlas on the individual space and used for further analysis^42^. The PLV (described in Lachaux et al.^64^) was applied to calculate functional connectivity on source signals in the DMN. The combination of wMNE and PLV was shown to precisely identify functional brain networks in the beta frequency band derived from scalp EEG^42,66^.

**Figure 3:**
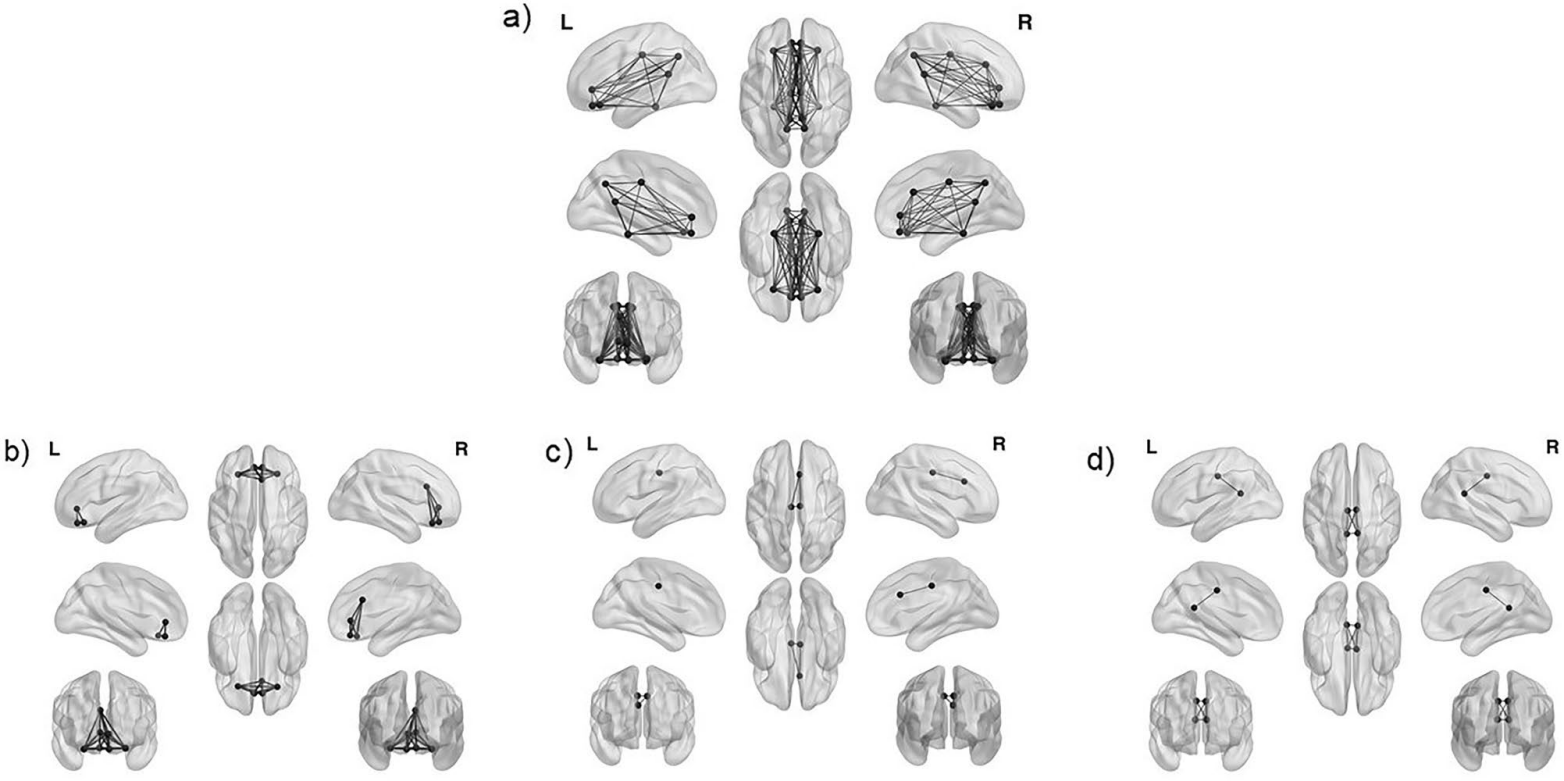
Analyzed subsystems of the Default Mode Network (DMN). (**a**) All 15 regions of interest of the DMN, (**b**) 7 regions of interest of the anterior DMN, (**c**) 3 regions of interest for the core DMN (**d**) 4 regions of interest for posterior DMN. The brain networks were visualized with BrainNet Viewer^69^.

The 15 ROI for the DMN were: left and right isthmus cingulate, left and right medial orbito-frontal, left and right posterior cingulate, left and right precuneus, left and right rostral anterior cingulate, left and right lateral orbitofrontal, left and right parahippocampal, right caudal anterior cingulate 2. The regions coordinates are presented in Supplementary Table S1. Moreover, fMRI studies observed age and AD-related changes of the DMN in subsystems, and we therefore subdivided it into anterior and posterior^16^ as well as core portions^21^ (see Fig. 3). The anterior DMN was defined as left and right rostral anterior cingulate cortex, left and right lateral orbito-frontal, left and right medial orbito-frontal, right caudal anterior cingulate cortex. The posterior DMN was defined as: left and right isthmus cingulate, left and right posterior cingulate cortex. The core of the DMN included the right caudal anterior cingulate cortex and the left and right posterior cingulate cortex.

The PLV was calculated for each of the 4 epochs, and the mean PLV of each epoch was averaged per subject. More specifically, the within-DMN PLV was calculated by averaging the pair-wise PLVs across all relevant pairs. The resulting connectivity matrices were then exported for beta frequency band (14–25 Hz)^42^ to MATLAB or SPSS.

### Frontal midline theta examination and power spectrum analysis

After excluding electrical bridges, raw data were band-pass filtered between 2 and 9 Hz and exported to EEGLAB 14.1.2^67^. Data was decomposed by using the AMICA algorithm^68^, which resulted in 128 independent components. Frontal midline theta components were defined and extracted as described in^38,57^. This resulted in one frontal midline theta component per participant, from which maximum power spectral density was extracted with the Welch Method (time window = 240 s, window length = 2 s). For dipole localization, the individual components were averaged over the whole sample. The average component was localized with a single dipole in a 3-shell-sphere head model.

### Graph theoretical analysis

Functional network characteristics of the DMN, based on the weighted and undirected PLV matrices, were calculated with algorithms of the Brain Connectivity Toolbox^69^. Strength was characterized as the sum of weights of edges connected to a node. An average was computed for the whole DMN and each subsystem. To evaluate strength, an absolute threshold was applied to the connectivity matrix at t = 0.95. The results were exported to SPSS for statistical analyses.

### Neuropsychological tests

Executive functions were assessed with the response inhibition task (INHIB, reaction time and percentage correct responses), and the Trail Making Test parts A and B (time to complete the test). Working memory was measured with the Corsi-Block Tapping Task forward (visual-spatial short term memory) and backward (visual-spatial working memory). All tests were implemented in the automated computer-based Vienna Test Battery 6.82.000 (Schuhfried GmbH, Mödling, Austria).

### Statistics

SPSS 26 for Windows (IBM, Armonk, NY, United States) was used for all statistical analyses. Spearman-rank correlations were used for all correlation analyses to account for non-normal distributed variables and outliers. The Kruskal–Wallis-Test was used for detecting group differences. A significance level of p = 0.05 was set for all analyses. Type 1 errors were corrected with Bonferroni.

## Supplementary Information

Supplementary Information.

## Data Availability

The data that support the findings of this study are available on reasonable request from the corresponding author (CR).
