# Publisher Correction: Source connectivity patterns in the default mode network differ between elderly golf-novices and non-golfers

**DOI:** 10.1038/s41598-023-34581-2

**Published:** 2023-05-05

**Authors:** J. K. Gowik, C. Goelz, S. Vieluf, F. van den Bongard, C. Reinsberger

**Affiliations:** Department of Exercise and Health, Institute of Sports Medicine, Paderborn University, Warburger Straße 100, 33098 Paderborn, Germany

Correction to: *Scientific Reports* 10.1038/s41598-023-31893-1, published online 17 April 2023

The original version of this Article contained an error in the order of the Figures. Figure 1 was published as Figure 2, Figure 2 was published as Figure 3, and Figure 3 was published as Figure 1. The Figure legends were correct from the time of publication.

The original Figures 1, 2 and 3 and accompanying legends appear below for the record.

**Figure 1:**
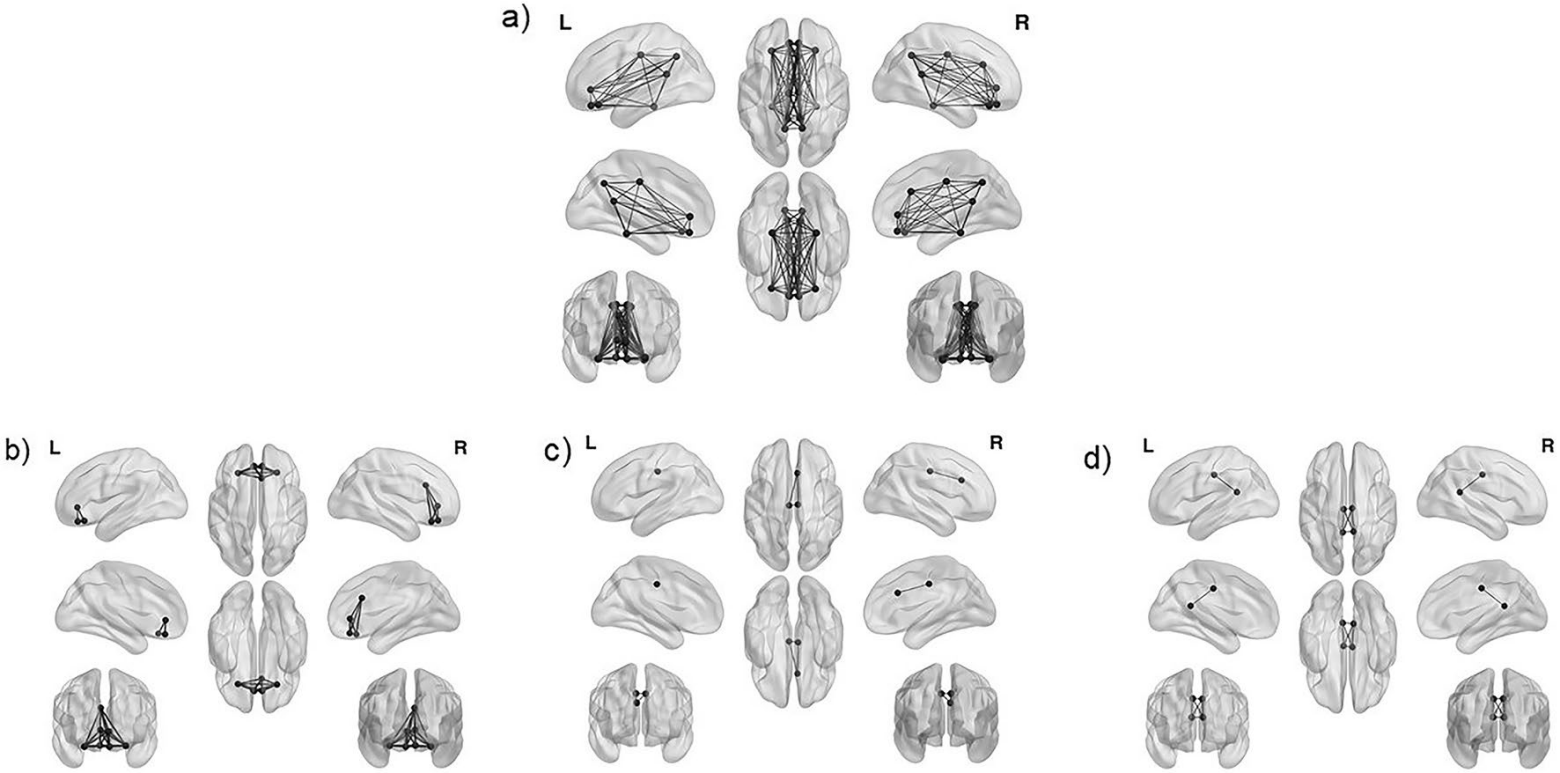
Localization of frontal midline theta (FMT, 4–7 Hz) based on the 22 individual components. MNI coordinates: X = 4.2 Y = 7.9 Z = 30.8. The brain networks were visualized with the BrainNet Viewer^70^.

**Figure 2:**
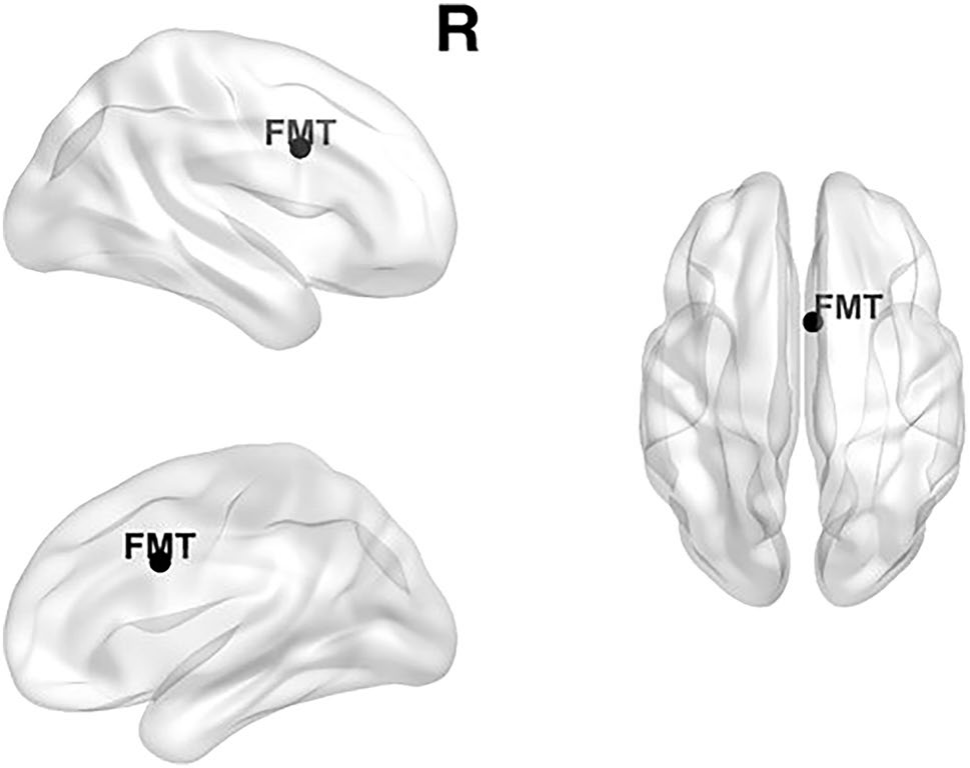
Data processing stream overview adapted from Kabbara et al.^41^. Blue box: Determination of frontal midline theta with independent component analysis. The average component was localized within Brodman Area (BA) 24. Power spectrum density (Welch method) was used to extract relative power over the whole epoch. Orange box: Preprocessing steps and selection of epochs for source connectivity and power analysis in the Default Mode Network (DMN). Green box: Preprocessing of native MRI and co-registration with the electrode coordinates. *AMICA* adaptive mixture independent component analysis, *ICA* independent component analysis, *wMNE* weighted minimum norm estimation, *BEM* boundary element model, *PLV* phase locking value.

**Figure 3:**
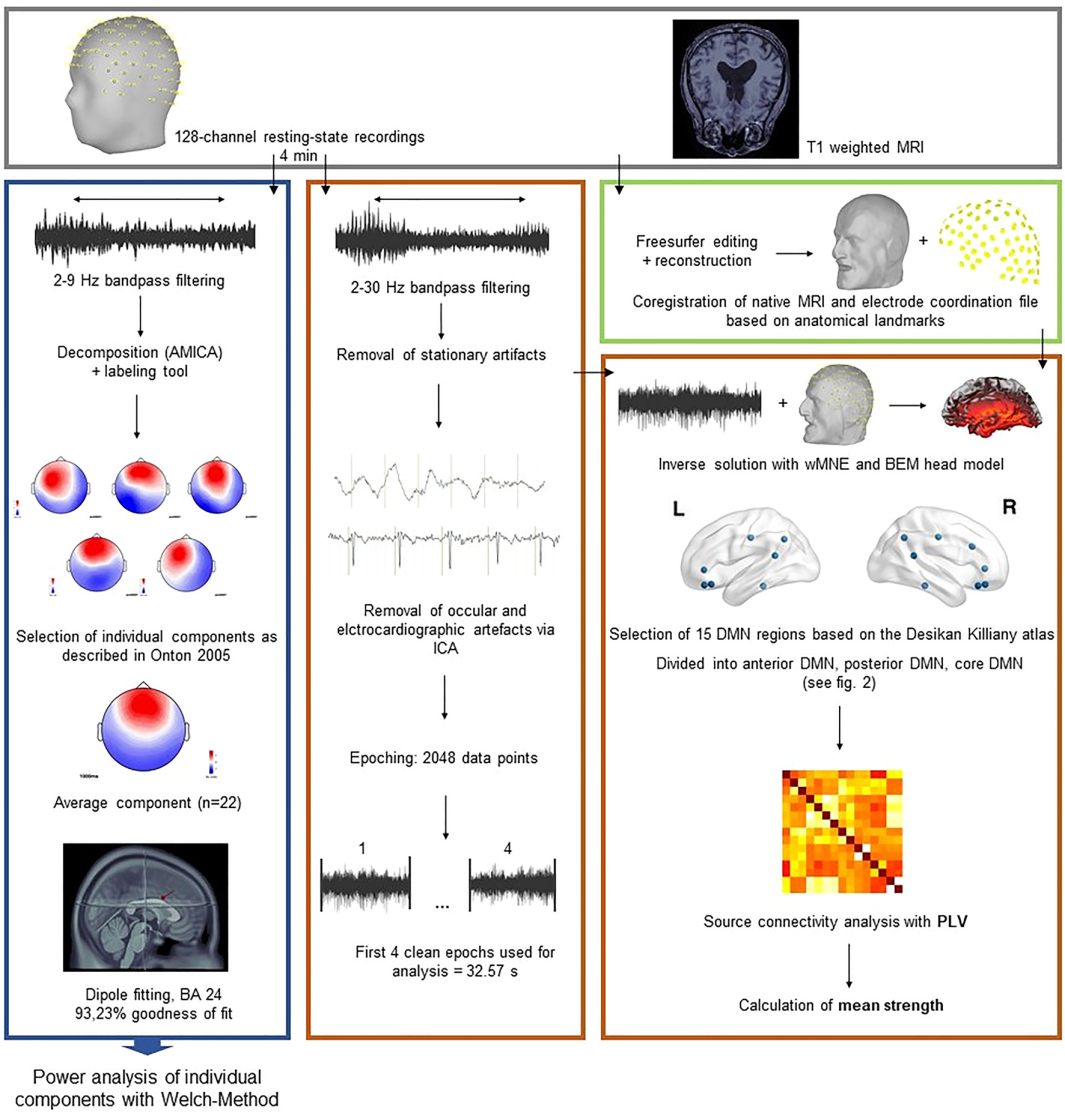
Analyzed subsystems of the Default Mode Network (DMN). (**a**) All 15 regions of interest of the DMN, (**b**) 7 regions of interest of the anterior DMN, (**c**) 3 regions of interest for the core DMN (**d**) 4 regions of interest for posterior DMN. The brain networks were visualized with BrainNet Viewer^69^.

The original Article has been corrected.

