# Supplementary material for: Source connectivity patterns in the default mode network differ between elderly golf-novices and non-golfers
Source: Sci Rep. 2023 Apr 17;13:6215. doi: 10.1038/s41598-023-31893-1 (PMC10110620; doi:10.1038/s41598-023-31893-1)
Supplement: Supplementary file 1 — Supplementary Information. [file 41598_2023_31893_MOESM1_ESM.docx]

**Source Connectivity Patterns in the Default Mode Network Differ Between Elderly Golf-Novices and Non-Golfers**

*JK Stroehlein, C Goelz, S Vieluf, F van den Bongard, C Reinsberger*

Supplementary Table 1.

Localization of DMN hubs based on the Desikan Killiany atlas:

| **DMN hub** | **x** | **y** | **z** |
| --- | --- | --- | --- |
| l. isthmuscingulate | -6.624240 | -47.248045 | 16.969356 |
| l.lateralorbitofrontal | -24.788431 | 28.715777 | -16.968762 |
| l. medialorbitofrontal | -5.406928 | 36.933371 | -18.001864 |
| l. parahippocampal | -23.907604 | -33.142327 | -19.249481 |
| l. posteriorcingulate | -5.701530 | -18.390072 | 38.473745 |
| l. precuneus | -9.690527 | -58.233298 | 36.662633 |
| l. rostralanteriorcingulate | -4.385862 | 37.523613 | -0.212297 |
| r. caudalanteriorcingulate | 5.012041 | 22.258100 | 27.639678 |
| r. isthmuscingulate | 7.091746 | -46.163380 | 16.740261 |
| r. lateralorbitofrontal | 24.236422 | 29.349355 | -17.996568 |
| r. medialorbitofrontal | 5.859795 | 37.568028 | -16.583859 |
| r. parahippocampal | 25.382203 | -33.021476 | -18.144719 |
| r. posteriorcingulate | 5.685813 | -17.196104 | 38.859022 |
| r. precuneus | 9.636033 | -57.310060 | 37.845502 |
| r. rostralanteriorcingulate | 5.367839 | 37.109173 | 1.676762 |
